# Anti-thrombotic agents derived from snake venom proteins

**DOI:** 10.1186/s12959-016-0113-1

**Published:** 2016-10-04

**Authors:** Tur-Fu Huang, Chun-Chieh Hsu, Yu-Ju Kuo

**Affiliations:** Institute of Pharmacology, College of Medicine, National Taiwan University, No.1, Sec. 1, Jen-Ai Rd, Taipei, Taiwan

**Keywords:** Snake venom proteins, Disintegrins, Antithrombotic agent, Arterial thrombosis, Angiogenesis, Septic inflammation

## Abstract

Snake venoms affect blood coagulation and platelet function in a complex manner. However, two classes of venom proteins, snaclecs and disintegrins have been shown to specifically target receptors including GPIb, α2β1, GPVI, CLEC-2 and integrins αIIbβ3, αvβ3, α5β1 expressed on platelets, endothelial cells, phagocytes, tumor cells, thus affecting cell-matrices and cell-cell interactions. Here, we focus on disintegrins, a class of low molecular mass Arg-Gly-Asp(RGD)/Lys-Gly-Asp(KGD)-containing, cysteine-rich polypeptide derived from various viper snake venoms. This review describes the potential applications of disintegrins in field of integrin-related diseases, especially arterial thrombosis, angiogenesis, tumor progression and septic inflammation. In addition, a novel RGD-containing disintegrin TMV-7 is being developed as a safer antithrombotic agent with minimal side effects, such as thrombocytopenia and bleeding.

## Background

Viperidae snake venoms profoundly affect blood coagulation and platelet function. Ouyang et al. [1] classified them into procoagulants, including factor X activator, prothrombin activator, thrombin-like enzymes and platelet aggregation inducer, and coagulation inhibitors, including fibrinogenolytic enzymes, prothrombin activation inhibitor, Factor X inhibitors, and platelet aggregation inhibitors. This review will focus on the active components that inhibit platelet function. Regarding platelet aggregation inhibitors, many venom components including ADPase, snake venom metalloproteinase (SVMP), phospholipase A2, GPIb and IX binding proteins (i.e. snaclec) and disintegrins have been reported to suppress platelet aggregation in vitro. However, snaclecs and disintegrins affect in vivo hemostasis and cell adhesion in a specific manner. Snaclecs can either induce platelet aggregation through vWF modulation, GPIb, α2β1, CLEC-2 and GPVI binding; or inhibit platelet aggregation via GPIb blockade. The biological activity of snaclecs has been reviewed comprehensively [2]. In the following, we will focus on disintegrins and their potential applications.

## Review

### Role of integrins in thrombosis and hemostasis

Integrins, a family of non-covalently linked α/β heterodimeric cell adhesion receptors, play vital roles in platelet aggregation, inflammatory reactions, cell adhesion, migration, angiogenesis and other biological processes [3].

Upon vascular injury, platelets adhere to the exposed endothelium through the recognition of adhesive proteins (i.e. vWF, collagen, fibronectin, laminin and fibrinogen) by their respective platelet membrane receptors (i.e. GPIb, α2β1/GPVI, α5β1, α6β1 or αIIbβ3). Nowadays, α2β1/GPVI are known to mediate collagen adhesion/activation of platelets, whereas αIIbβ3 mediates the adhesion of fibrinogen, and the subsequent platelet-platelet aggregation, a common final step of platelet aggregation shared all stimulating agonists [4, 5]. Generally, the activation of platelets consists of many signal transduction pathways including PLA2/PLC activation and the rise of cytosolic free Ca^2+^ after the ligation of agonist with its receptor, leading to shape change, release reaction of ADP and serotonin as well as thromboxane A2 formation, enhancement of procoagulation activity, and finally the activation of αIIbβ3. The binding of plasma fibrinogen to the activated αIIbβ3 bridges adjacent platelets and lead to platelet aggregation which is reinforced by the subsequent fibrin formation forming a hemostatic plug. However, pathological thrombi may be formed due to chronic endothelial injury in atherosclerotic patients [6].

### Disintegrins as anti-thrombotic agents

Trigramin, a non-enzymatic small molecular weight polypeptide, was first discovered in 1987 [7]. Our earlier studies showed that disintegrins derived from *T. gramineus*, *Agkistrodon halys*, and *Agkistrodon rhodostoma* [8–10] inhibited platelet aggregation elicited by various aggregation agonists including ADP, epinephrine, sodium arachidonate, collagen and Ca^2+^ ionophone A23187 with similar IC50 concentrations. They neither affect shape change nor the cAMP level. Further studies showed that trigramin and echistatin purified from *T. gramineus* and *Echis carinatus*, inhibited fibrinogen binding to ADP-stimulated platelets [7, 11]. ^125^I-trigramin showed less than 5 % binding to ADP-stimulated platelets in Glanzmann’s thrombasthenia patients (a genetic disease with αIIbβ3-defect) compared to normal platelets, suggesting that αIIbβ3 is the target of trigramin. In addition, 7E3 mAb raised against αIIbβ3 and RGDS inhibited ^125^I-trigramin binding. Subsequent sequencing of trigramin showed that it is a RGD-containing single polypeptide with 72 amino acid residues and six disulfide bonds. Reduced trigramin lost its inhibitory activity for platelet aggregation and binding capacity to platelets, indicating that the biological activity of trigramin depends upon the presence of the RGD sequence and its steric structure maintained by disulfide bridges [7, 12]. Upon intravenous administration, trigramin prolonged the bleeding time of severed mesentery arteries, further supporting its in vivo antithrombotic activity [13]. Later, other disintegrins, such as kistrin and applagin were also shown to prevent experimental thrombosis in dogs [14, 15]. Since then, many pharmaceutical companies have developed potential antithrombotic agents based on the structure of these disintegrins. Among these disintegrins, barbourin, a KGD-containing polypeptide, showed a higher specificity toward platelet αIIβ3 than to endothelial αvβ3 [16]. Thus, eptifibatide (Integrillin), a cyclic KGD-peptide, has been successfully developed as an αIIbβ3 antagonist, used clinically for prevention of restenosis during percutaneous transluminal coronary angioplasty (PTCA) [17]. Tirofiban is also designed to mimic the RGD-loop structure of the disintegrin, echistatin [18]. In contrast to the first anti- aggregation agent, the chimeric monoclonal c7E3Fab (Abciximab, Reopro) a mAb raised against αIIbβ3 [19], tirofiban and eptifibatide are small-mass αIIbβ3 antagonists derived from snake venom disintegrins. Vipegitide, a folded KTS-containing peptidomimetic molecule, inhibited the adhesion of α1/α2 integrin toward collagen, and ADP and collagen-I induced platelet aggregation in PRP and whole human blood [20], providing strategy for developing α2β1 antagonists as antiplatelet agents.

### Disintegrins as an anti-tumor and anti-angiogenesis agents

Integrin αvβ3 modulates adhesion, migration and proliferation of endothelial cells, smooth muscles, fibroblasts and transformed cells, and thus plays crucial roles in angiogenesis, restenosis, tumor cell migration and atherosclerosis [21]. It has been reported that disintegrins inhibit adhesion of tumor cells and endothelial cells to extracellular matrix (ECM) through αvβ3 and α5β1. Trigramin inhibited human melanoma cells adhesion and spreading on fibronectin and fibrinogen [22], and trifavin inhibited lung colonization of B16F10 melanoma cells in an experimental model [23].

We previously reported that some disintegrins (accutin, rhodostomin) dose-dependently inhibited the adhesion of endothelial cells to ECM, cell proliferation, matrigel-induced capillary tube formation, and neovascularization in a chick chorioallantoic membrane (CAM) model, mainly through αvβ3 blockade [24]. Accutin is the first disintegrin reported to induce apoptosis of HUVECs [25]. Rhodostomin caused a higher percentage of cells at G2/M phase, the cleavage of β-catenin, and poly (ADP-ribose) polymerase during apoptosis [26]. Contortrostatin, a dimeric disintegrin that binds to αvβ3, αvβ5 and/or α5β1, was shown to inhibit the adhesion and invasion of tumor cells and endothelial cells in vitro. In an orthotopic xenograft model in nude mice, contortorstatin displayed good anti-tumor and antiangiogenic potential against a breast cancer cell line (MDA-MB-435) [27]. Vimocin and Vidapin, two synthetic peptides derived form KTS-containing disintegrins obtustatin and viperistatin, were shown to inhibit angiogenesis induced by VEGF and glioma in CAM assay, suggesting that they may serve as dual α1β1/α2β1 integrin antagonists in anti-angiogenesis and cancer therapy [28]. Cilengitide, an RGD-containing pentapeptide and other αv integrin inhibitors have entered phase II/III clinical study as monotherapy for different tumor types. However, Cilengitide did not alter the pattern of glioblastoma progression [29], and in a phase III clinical trial, the addition of Cilengitide to standard care did not improve overall survival in patients with newly diagnosed glioblastoma [30]. There are still some on-going phase I-II clinical trials in combinative therapy for glioblastoma and non-small-cell lung cancer [31, 32].

### Disintegrins as anti-inflammatory agents

The innate immune system is the first line of defense against microbial pathogens, protecting the host from infection. In humans, Toll-like receptors (TLRs) specifically recognize different microbial patterns to initiate signaling pathways leading to inflammation [33]. TLR4 recognizes lipopolysaccharide (LPS) -containing Gram negative bacteria while TLR2 recognizes peptidoglycan of Gram positive bacteria [34]. The β2 and β3 integrins regulate leukocyte trafficking and function. Vitronectin and αvβ3 have roles in initiating TLR2 responses to bacterial lipopeptide [35]. Our recent studies showed that the disintegrin rhodostomin (Rn) possesses anti-inflammatory activity mainly through blocking αvβ3-induced NFkB and MAPK pathways, and MyD88-dependent TLRs (including TLR2 and 4) in the production of cytokines in phagocytes. Thus, Rn suppresses cytokine release, inhibits cell adhesion and migration in vitro and even attenuates the acute inflammatory activity in mice caused by bacterial infections [36, 37]. The activation of coagulation leading to microvascular thrombi causes multiple organ failure and correlates with mortality in severe sepsis [34]. Rn was also shown to increase significantly the survival rate of septic mice [36, 37]. The antiplatelet activity of Rn through αIIbβ3 blockade may be partially responsible for its capacity to reduce thrombi formation. The potential application of rhodostomin as an inhibitor of TLR-2 and TLR-4 activation provides a promising lead for drug development in infectious diseases induced by complicated microbial patterns.

### Novel αIIbβ3 antagonists derived from disintegrins

Although clinically available αIIbβ3 antagonists are highly efficacious antithrombotics, their uses are currently limited to percutaneous coronary intervention (PCI) due to adverse reactions of thrombocytopenia and bleeding. Increased bleeding risk prevents this class of αIIbβ3 antagonists from being used at higher doses and in patients at risk of bleeding [38]. It has been recognized that binding of RGD-mimetic drugs including tirofiban and eptifibatide to the RGD recognition site of αIIbβ3 induces conformational changes and exposure of αIIbβ3 neoepitopes (called ligand-induced binding sites, LIBs) which are recognized by several murine monoclonal antibodies [39]. As a consequence, patients with preformed antibodies against LIBs may engage the integrin β3 subunit such that FcγRII and its downstream signaling pathways become activated, resulting in platelet clearance from circulation and concomitant thrombocytopenia [40, 41]. Thus, it is crucial to develop novel αIIbβ3 antagonists that do not cause thrombocytopenia or bleeding. Recently RUC-1,2 and 4 compounds have been shown to be efficacious αIIbβ3 antagonist with minimal effect in altering the αIIbβ3 conformation [42, 43]. On the other hand, a membrane permeable compound, mp6, was shown to inhibit the outside-in signaling without affecting the fibrinogen-αIIbβ3 ligation [44]. In our laboratory, we found a unique RGD-containing disintegrin, TMV-7, purified from *T. macrosquamatus* venom, which like RUC-2, had a minimal priming effect in induction of fibrinogen-binding or PAC-1 binding, reflecting that TMV-7 induces little exposure of LIBs (unpublished data). It binds preferably to αIIb, a binding epitope different from those of mAb 7E3, tirofiban, eptifibatide and most RGD-containing disintegrins including dimeric disintegrin, and short- and medium-size disintegrins. TMV-7 has also been shown to be an efficacious antithrombotic agent in FeCl_3_-induced carotid artery injury, and irradiation-induced mesenteric thrombosis models. At effective doses, TMV-7 did not significantly prolong the bleeding time. Its unique mechanism of action may be related to inhibiting outside-in signaling without affecting talin-mediated inside-out signaling and clot retraction. Recent studies show that Gα13 and talin play critical roles in thrombin-induced integrin bidirectional signaling and bind to mutually exclusive but distinct sites within integrin β3 cytoplasmic domain in opposing waves, suggesting that targeting outside-in signaling may prevent thrombosis without affecting physiological hemostasis [45–47]. Therefore, the elucidation of the structure-activity relationship between TMV-7 and αIIbβ3 on a molecular level may provide clues for drug development of an ideal antithrombotic RGD-mimetic with a better safety profile.

### Translational medicine derived from disintegrins

Over the last three decades, extensive researches on RGD/KGD-containing disintegrins focused on their interaction with platelet αIIbβ3 and endothelial or tumor αvβ3, resulting in the successful development of the efficacious αIIbβ3 antithrombotic agents such as tirofiban and eptifibatide. However, the efforts for application of αvβ3-specific RGD-mimetics in tumor therapy are still going-on. The possible potential use of αvβ3 disintegrin in septic inflammation is worthy of further investigation. However, the possible antigenicity and brief half-life of intact disintegrins in circulation limit their direct ultilization as therapeutic agents although their molecular masses are usually around 4000 ~ 7000 daltons. A alternative strategy in developing these disintegrins may be approached by PEGylation or conjugation with human serum albumin to minimize antigenicity or prolong their half-lives. A PEGylated αIIbβ3 disintegrin (PEGylated rhodostomin) has been reported to have an improved antithrombotic activity [48]. PEGylated rhodostomin (PRn) has higher antithrombotic potency and a longer half-life in vivo compared with native rhodostomin. In addition, PRn shows a better safety profile at an effective dose in vivo. Therefore, PEGylation may be one ideal option in modifying disintegrin derivatives to produce a safe therapeutic agent.

## Conclusions

The discovery of the naturally occurring disintegrins has inspired much research into the molecular interaction of RGD/KGD disintegrins with integrin αIIbβ3, αvβ3 and other integrins, leading to drug development of potential agents in the fields of arterial thrombosis, angiogenesis, tumor metastasis, inflammation and other integrin-related diseases. With the aid of advanced molecular biology techniques and the elucidation of physiological and pathological roles of integrins, these disintegrins and their mutants, targeting the binding site of the specific integrin would be helpful for the further dissection of their efficacy and adverse reactions mechanisms.

X-ray crystallography, ligand-receptor docking and bioinformatics regarding the binding ligands (disintegrin and mutants) toward integrins, and their atomic interactions should accelerate the discovery of novel therapeutic agents, especially the small-mass RGD-mimetics derived from the naturally-occurring disintegrins.

## Abbreviations

ADP: Adenosine diphosphate
ECM: Extracellular matrix
KGD: Lys-Gly-Asp
LIBs: Ligand-induced binding sites
LPS: Lipopolysaccharide
mAb: Monoclonal antibody
PCI: Percutaneous coronary intervention
PEG: Polyethylene glycol
PLA2: Phospholipase A2
PTCA: Percutaneous transluminal coronary angioplasty
RGD: Arg-Gly-Asp
SVMP: Snake venom metalloproteinase
TLR: Toll-like receptor
vWF: von Willebrand factor
